# Conducting polymer PPy nanowire-based triboelectric nanogenerator and its application for self-powered electrochemical cathodic protection†
†Electronic supplementary information (ESI) available: Microscope images, OCP variation and Tafel curves of Q235 carbon steel which is connected to the cathodic protection system driven by W-TENG are reported here. See DOI: 10.1039/c6sc02562e



**DOI:** 10.1039/c6sc02562e

**Published:** 2016-06-27

**Authors:** Siwen Cui, Youbin Zheng, Jun Liang, Daoai Wang

**Affiliations:** a State Key Laboratory of Solid Lubrication , Lanzhou Institute of Chemical Physics , Chinese Academy of Sciences , Lanzhou 730000 , China . Email: wangda@licp.cas.cn; b University of Chinese Academy of Sciences , Beijing , 100049 , China

## Abstract

As a new type of energy harvesting device, the triboelectric nanogenerator (TENG) can convert almost all kinds of mechanical energy into electricity based on the coupling of triboelectrification and electrostatic induction. Here, a novel TENG is constructed with a conducting polymer polypyrrole nanowire (PPy NW) electrode, which is prepared by an electrochemical polymerization method with anodic aluminum oxide (AAO) as the template. The PPy NW-based TENG shows high output performance with a maximum short circuit current density of 23.4 mA m^–2^ and output voltage of 351 V, which can light 372 commercial red LEDs. Moreover, a self-powered anticorrosion system powered by the PPy NW-based TENG is designed, which can provide extra electrons to inject into the surface of the protected metals, forming effective impressed current cathodic protection by harvesting mechanical energy or wind energy. This smart device has potential applications for protecting metals from corrosion in daily life, industrial production and ocean exploration by harvesting the energies in the ambient environment.

## Abstract 2

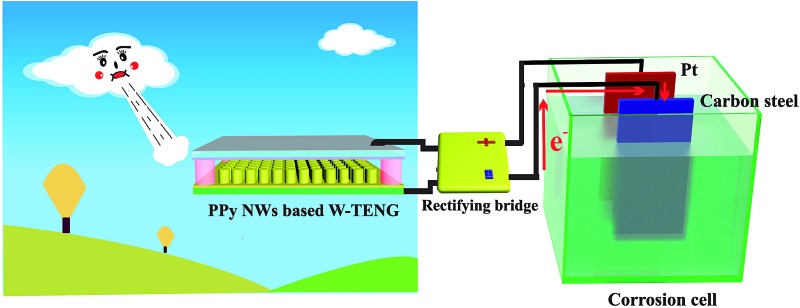
A novel triboelectric nanogenerator (TENG) was constructed with PPy nanowires (PPy NWs). By harvesting the energies in the ambient environment, the PPy NW-based TENG can act as a power supplier and provide extra electrons to the surface of the connected metals, forming effective impressed current cathodic protection.

## Introduction

With the increasingly serious energy crisis and environmental pollution, developing green and renewable energies has drawn the widespread attention of the world.^1–3^ Harvesting energies from the ambient environment would be one of the most promising approaches to relieve the threat of energy depletion.^4,5^ Recently, the triboelectric nanogenerator (TENG), a new type of mechanical energy harvesting device invented by Wang and co-workers in early 2012,^6^ has been widely studied to produce electricity by harvesting almost all kinds of mechanical energy that exist in our daily life, such as human motion,^7^ vibration energy,^8,9^ wind energy,^10,11^ water wave energy,^12,13^ air-flow energy,^14^ rain drops,^15^ sound energy,^16^ *etc.*

The working principle of the smart TENG is based on the coupling effect of triboelectrification and electrostatic induction during the contact or friction process between two triboelectrode materials with opposite triboelectric polarities.^17^ Since triboelectrification is a surface charging effect, the surface structures and compositions of triboelectrode materials are the crucial factors to determine the output of TENGs.^18^ Although virtually all materials exhibit the triboelectricity property, it is still a challenge to select and develop new triboelectrode materials with special micro- and nanostructures to further improve the output of TENGs.^19,20^ Till now, several types of materials, including insulated polymers such as polytetrafluorethylene (PTFE), nylon, polydimethylsiloxane (PDMS), *etc.*,^21,22^ inorganic semiconductors (TiO_2_, ZnO, *etc.*),^23,24^ and metals (Au, Al, *etc.*),^25,26^ were used as the triboelectrode materials in TENGs. Among the conducting polymers, the conducting polymer polypyrrole (PPy) has attracted much more attention in the fields of batteries, supercapacitors, sensors, electrochemical actuators and biochemistry, owing to its high electric conductivity, flexibility, low cost, stability, biocompatibility, and good redox property.^27–31^ In addition, PPy with one dimensional nanostructures can be easily synthesized by controlling the chemical or electrochemical polymerization conditions.^32,33^ The π electrons in the PPy molecules, similar to the free electrons in metal conductors, are able to move along the carbon chain with double bond alternation in the conjugated polymer backbone.^34,35^ Furthermore, PPy has high specific capacitance and a fast redox reaction rate, resulting in rapid charge storage.^36^ As a consequence, PPy could be a potential promising triboelectrode material for a high output TENG. Besides, owing to the nature of the semiconductor properties, PPy was first reported in the formation of a TENG by Wang and co-workers, which could replace the conventional metal electrode materials.^37,38^

Recently, TENGs were widely studied for powering portable electronics^7,39,40^ and self-powered devices.^41–43^ As a new potential application, it has been also introduced in the cathodic protection system for protecting metals from corrosion,^15,44,45^ which is a common phenomenon and results in huge economic losses and security risks in modern society.^46,47^ Remarkably, the TENG could harvest available energies from the ambient environment, such as wave energy, mechanical energy, wind and rain drops, to form a self-powered impressed current cathodic protection system. Metals could be effectively protected from corrosion when they are connected to the self-powered cathodic protection system driven by the TENG. Traditional electrochemical cathodic protection has two types, *i.e.*, impressed current cathodic protection^48^ and sacrificial anode cathodic protection.^49^ However, either the external power source or the consumption of active anode metals limits their applications and causes environmental problems. In particular, the impressed current cathodic protection system is also restricted by the environment in which it is used and the power supply unit. Therefore, it is necessary to develop a novel and smart cathodic protection system without external electric energy and loss of materials. The TENG-powered cathodic protection system could provide a new way to partly solve these problems.

Herein, an interesting impressed current cathodic protection system powered by a PPy nanowire (PPy NW)-based TENG was designed. The PPy NWs were prepared by an electrochemical polymerization method with anodic aluminum oxide (AAO) as the template, and then assembled with a polyvinylidene fluoride (PVDF) triboelectrode to form the TENG. In this device, the conducting polymer of PPy on Ti, which are both resistant materials, is chosen to replace conventional metal materials with poor anticorrosion properties for the TENG. The PPy nanowire structure with a diameter of 100 nm could significantly increase the frictional contact area to enhance the output performance of the TENG. On the other hand, PVDF with excellent processability and flexibility is chosen as the counter triboelectrode to make it easier to collect the weak energies from the ambient environment. Here, TENGs with two different kinds of structure for harvesting mechanical energy or wind energy, respectively, have been designed to generate electricity, and then they were used to power a cathodic protection system to protect metals from corrosion.

## Results and discussion

**Fig. 1:**
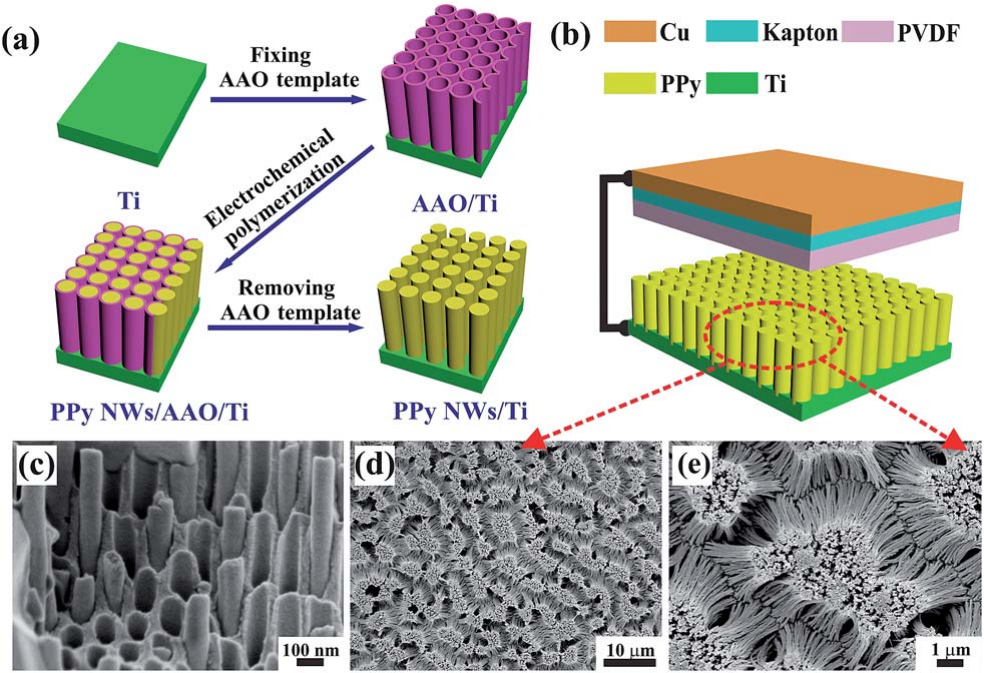
(a) Schematic depiction of PPy NW preparation by electrochemical polymerization with AAO as the template. (b) Schematic illustration of the designed PPy NW-based TENG. (c–e) FESEM images of PPy NWs before and after dissolving the AAO template.

Fig. 1a shows the schematic preparation of PPy NWs by an electrochemical polymerization process with AAO as the template. Firstly, the AAO template was fixed tightly on the surface of Ti substrate with epoxy resin. Then, PPy NWs were grown along the AAO pores by an electrochemical polymerization method. Finally, the AAO template was dissolved in NaOH solution to obtain the PPy NWs on Ti substrate. The FESEM images of the PPy NWs before and after dissolving the AAO template are shown in Fig. 1c–e. It can be seen that the PPy NWs have grown along the pores of the AAO template, and the diameter of the PPy NWs is about 100 nm, matching well with the size of the AAO nanopores in Fig. 1c. The length of the PPy NWs is about 10 μm for electrochemical polymerization for 1 min at 3 V, and could be well controlled by adjusting the polymerization time. The PPy NWs are vertically aligned on the Ti substrate after dissolving the AAO template, as shown in Fig. 1d and e.

Fig. 1b shows the structure of the PPy NW-based TENG. Basically, there are two friction electrode materials with opposite triboelectric polarities in the TENG. When they are pressed into contact, the one with the higher tendency to lose electrons would be positively charged, while the other one would be negatively charged through accepting electrons. As shown in Fig. 1b, the PPy NWs on Ti substrate and PVDF spin-coated on Kapton film with Cu tape on the back are used as the triboelectrodes, respectively. The PPy material as an electron donor can easily provide electrons in its conjugated backbone, and the PVDF would act as an electron acceptor during the friction process. Thus, a simple TENG is constructed from these two materials with opposite triboelectric polarities and operated in a vertical contact and separation mode by a linear mechanical motor.

**Fig. 2:**
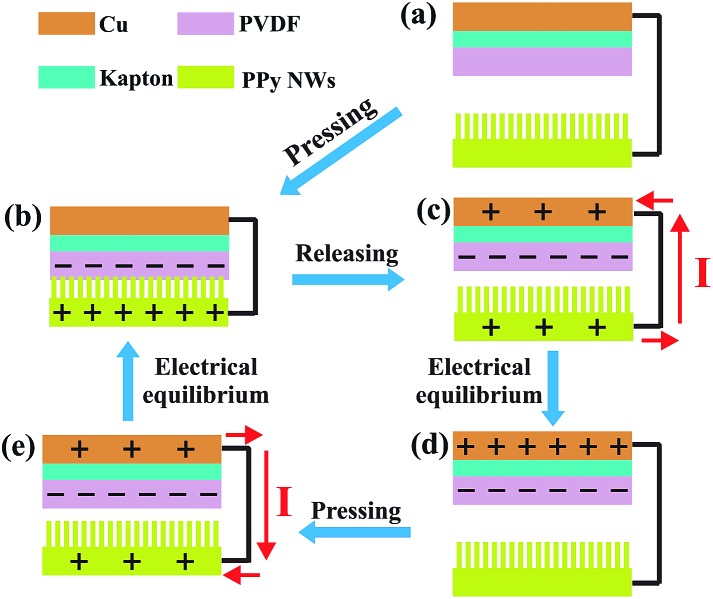
Schematic illustration of the proposed working principle of the PPy NW-based TENG.

The working principle of the PPy NW-based TENG in the vertical contact-separation mode is shown in Fig. 2, which is the coupling of triboelectrification and electrostatic induction. Before contact, there is no electron flow in the external circuit (Fig. 2a). Once an external force is applied to make the PPy NWs and PVDF contact, electrons of PPy will be injected into PVDF, resulting in positively charged PPy NWs and negatively charged PVDF (Fig. 2b). When the external force is released, the back electrode (Cu layer) generates the opposite charges to the PVDF layer due to the electrostatic induction effect, and an electric potential difference established between these two electrodes makes the electrical current flow from the PPy NW electrode to the Cu electrode (Fig. 2c). When the accumulated charges reach an electrical equilibrium, no current flow exists in the external circuit (Fig. 2d). However, once the device is repressed again by external force, the electric potential difference starts to reduce because the distance between the two electrodes becomes shorter. As a result, the electric current flows in a reverse direction from the Cu electrode to the PPy NW electrode (Fig. 2e) till a new electrical equilibrium is achieved (Fig. 2b).

**Fig. 3:**
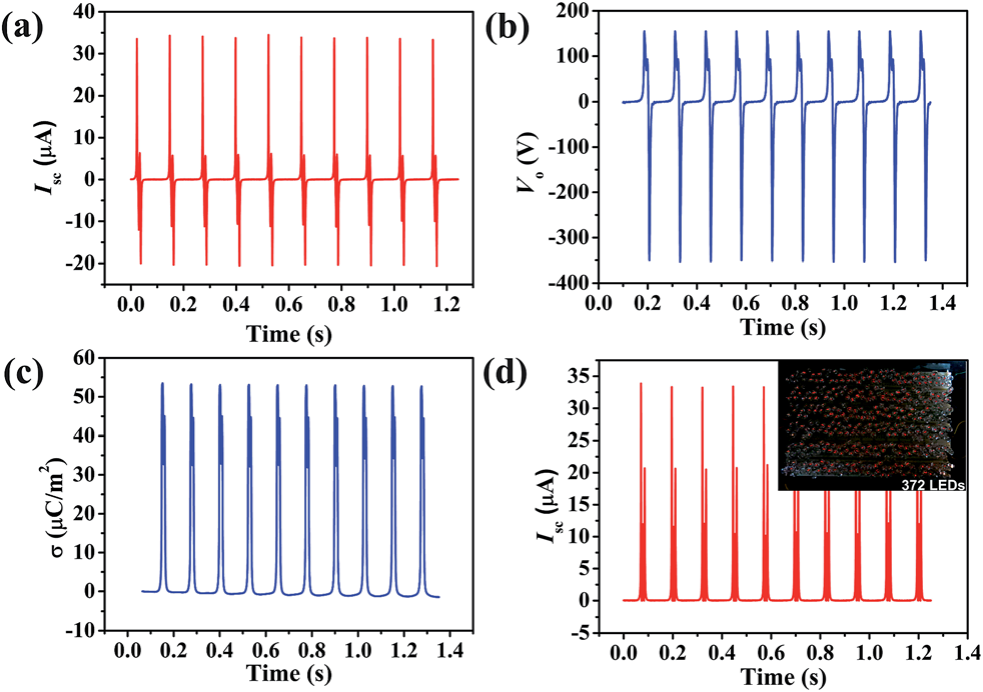
(a) Short circuit current (*I*_sc_), (b) output voltage (*V*_o_), and (c) charge density (*σ*) of the PPy NW-based TENG. (d) *I*_sc_ of the PPy NW-based TENG in one direction after rectification. The inset is the photograph of 372 commercial LEDs powered by the PPy NW-based TENG.

The output performance of the PPy NW-based TENG with a contact area of 12 cm^2^, including short circuit current (*I*_sc_), output voltage (*V*_o_) and charge density (*σ*), has been measured as shown in Fig. 3a–c. Since the PPy NW-based TENG is operated by repetitively pressing and releasing using a linear motor at a frequency of 8 Hz, the output signals show periodic changes. An instantaneous current would emerge to balance the generated triboelectric potential difference under the short circuit condition of the TENG. The peak value of *I*_sc_ reaches 33.7 μA. The *I*_sc_ exhibits alternating current (AC) behaviour, with an equal amount of electrons flowing in the opposite directions within one cycle. The *V*_o_ was measured with a load resistor of 100 MΩ and the maximum peak value was about 351 V. The surface charge density (*σ*), which is a key factor to determine the performance of the TENG, has been taken as a standard to evaluate the availability of a material for a TENG.^19,50^ The *σ* in Fig. 3c is the time integral of current and its stable peak value reaches around 53.1 μC m^–2^, which is considerably high for powering some small portable electronics and sensors. In addition, to obtain the current in one direction, a rectifying bridge was connected to the TENG and the *I*_sc_ is shown in Fig. 3d. After rectification, the PPy NW-based TENG can directly light 372 commercial red LEDs, as shown in the inset of Fig. 3d.

**Fig. 4:**
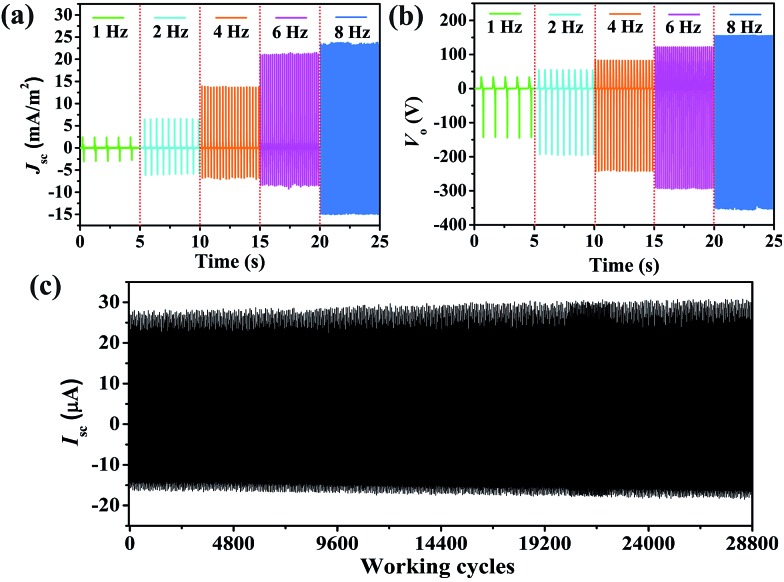
(a) *I*_sc_ and (b) *V*_o_ of the PPy NW-based TENG in the frequency range from 1 Hz to 8 Hz. (c) *I*_sc_ of the TENG working continuously for 28 800 cycles.

It is necessary to study the relationship between the output signals and various conditions because the mechanical energies harvested from the ambient environment are diverse and irregular.^51^ Here, we utilized classic periodic impacts to characterize the output performance of the PPy NW-based TENG. The frequency varied from 1 Hz to 8 Hz. The *I*_sc_ and *V*_o_ are plotted in Fig. 4a and b. It can be clearly seen that, as the frequency increased, both *I*_sc_ and *V*_o_ became larger. When the frequency is above 8 Hz, the output reaches the maximum values. For practical applications, the stability of the TENG is rather important for continuously harvesting energy from the ambient environent. Herein, a durability test has also been carried out under 8 Hz contact frequency with the TENG continuously working for about 28 800 cycles. The result is shown in Fig. 4c. After continuously working for 28 800 cycles, the *I*_sc_ of the TENG remains at a relatively stable value, indicating that the PPy NW-based TENG has good durability, which can provide a reliable guarantee for its possible application.

**Fig. 5:**
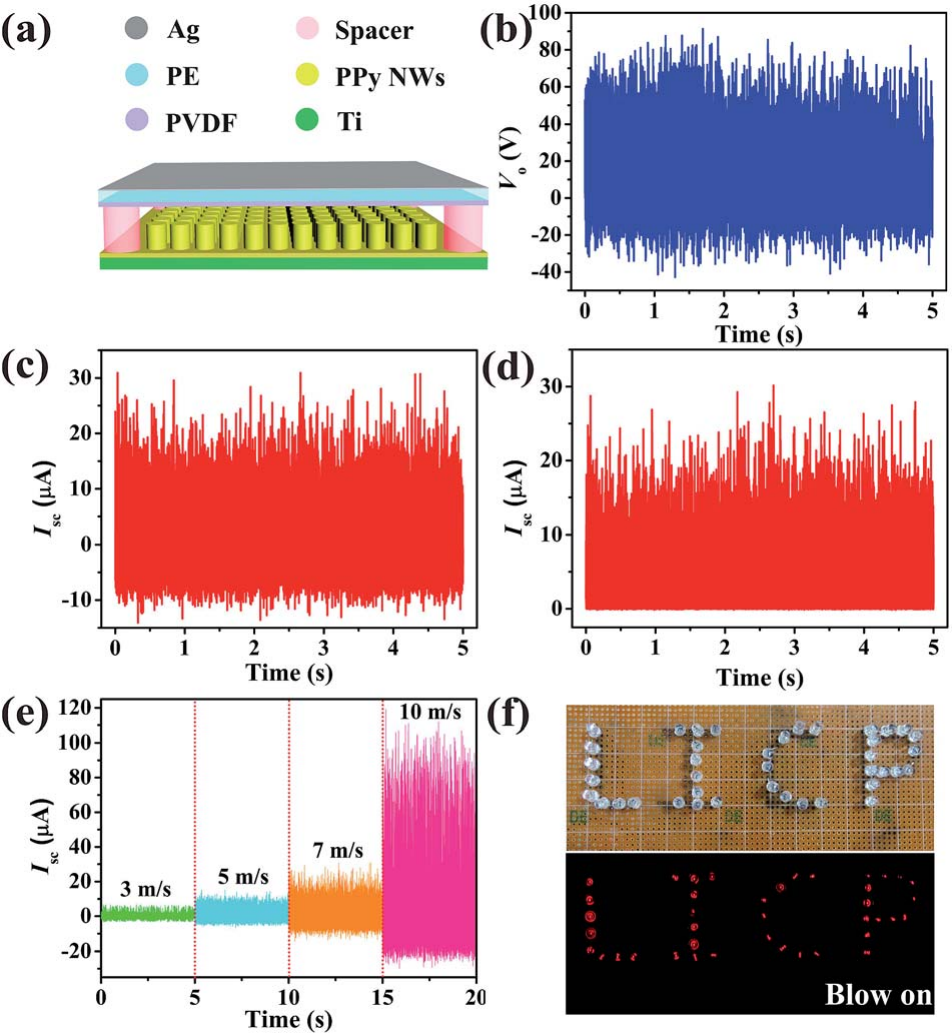
(a) Device structure of the designed PPy NW-based W-TENG. (b) *V*_o_, (c) *I*_sc_ and (d) the rectified *I*_sc_ at a wind speed of 7 m s^–1^ (on the scale of 4 BF). (e) The *I*_sc_ at different wind speeds from 3 m s^–1^ (2 BF) to 10 m s^–1^ (5 BF). (f) Photographs of 40 LEDs before and after being powered by the W-TENG.

To design a TENG for harvesting wind energy (W-TENG), a more flexible and transparent polyethylene (PE) thin-film (thickness of 10 μm) was introduced instead of Kapton as the substrate of the triboelectrode. As shown in Fig. 5a, PVDF was deposited on one side of the PE film by the electrospinning method while a thin Ag layer was sputtered on the other side. Then, the flexible PVDF-based triboelectrode was fixed on the four edges of the PPy NW-based triboelectrode with acrylic sheet (thickness of 1 mm) as a spacer. When the wind blows, the flexible PVDF based triboelectrode would strongly vibrate with high frequency. The effective and rapid switching between contact and separation of the two triboelectrodes is vital to determine the electrostatic potential, which is the driving force for the free electrons. In Fig. 5b–d, the electrical output measurement of the W-TENG was carried out at a wind speed of 7 m s^–1^. The maximum peak value of *V*_o_ reaches 90 V. During the triboelectrodes periodically contacting and separating from each other, the *I*_sc_ exhibits AC output with a peak value of 30 μA. The AC output is also rectified to be in one direction by a full-way rectifying bridge in Fig. 5d, which can light dozens of commercial LEDs, as shown in Fig. 5f. To investigate the relationships between the outputs of the W-TENG and wind speeds, the measurement was performed at different wind speeds from 3 m s^–1^ (2 BF on the Beaufort wind force scale) to 10 m s^–1^ (5 BF). The results in Fig. 5e show that the peak value of *I*_sc_ presents an obvious increase with the wind speed being raised, because the greater wind speed results in a higher vibration frequency and then the output would be enhanced. This W-TENG can generate electricity even at relatively low wind speeds owing to the high flexibility of the PVDF/PE triboelectrode.

Electrical energy generated by TENGs, a newly developed green energy, has great application prospects to meet energy needs in the future. Remarkably, TENGs serving as a power source can be used to protect metals from corrosion by the cathodic protection effect. Here, the cathodic protection anti-corrosion systems powered by the TENG driven by mechanical energy and the W-TENG driven by wind energy, with different structures, were investigated. For the first type of TENG as shown in Fig. 1b, it was driven by a linear mechanical motor, simulating mechanical energy. According to the previous report, the working frequency of a TENG is an important impact parameter during cathodic protection measurements. It is proven that a better cathodic protection effect is realized under a higher working frequency, resulting from the increase of the transferred charges.^44^ So, the cathodic protection measurement was carried out with a linear mechanical motor with a high working frequency of 8 Hz.

**Fig. 6:**
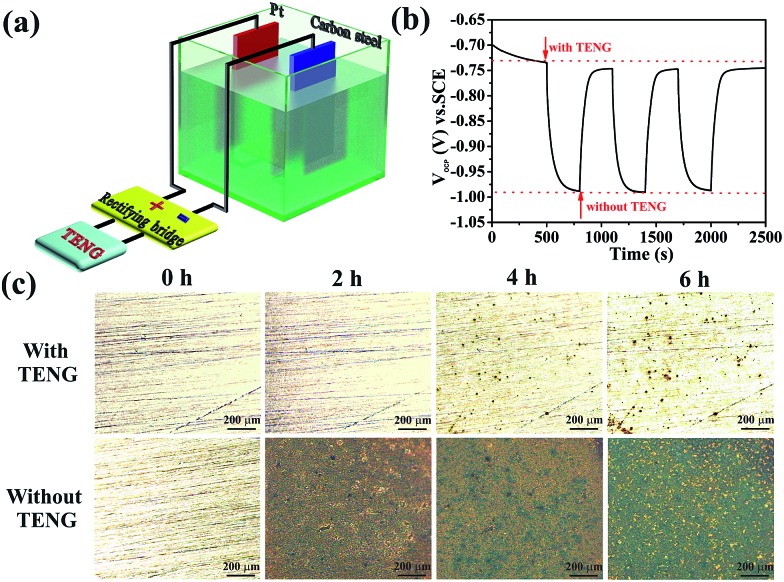
(a) Schematic diagram of the PPy NW-based TENG used for the cathodic protection of Q235 carbon steel in 3.5 wt% NaCl solution. (b) OCP changes of Q235 carbon steel coupled with and without the PPy NW-based TENG. (c) Microscopy images of the Q235 carbon steel immersed in 3.5 wt% NaCl solution for 2 h, 4 h and 6 h, separately, connected with (the top views) and without the PPy NW-based TENG (the bottom views).

Fig. 6a shows the design of the PPy NW-based TENG used for the cathodic protection system. The TENG is connected to a rectifying bridge, and a carbon steel is directly linked to the negative electrode of the rectifying bridge with a Pt electrode connecting to its positive electrode. Thus, the electrons generated by the TENG would be injected into the surface of the protected carbon steel to restrict corrosion. In a cathodic protection system, the open circuit potential (OCP) drop, as an important parameter, has been used to evaluate the cathodic protection efficiency. A more negative OCP shift indicates a better cathodic protection effect.^52^ As shown in Fig. 6b, the OCP variation of the carbon steel with and without the TENG are measured in a three-electrode system with a saturated calomel electrode (SCE) as the reference electrode and Pt as the counter electrode in a 3.5 wt% NaCl solution. The OCP of the carbon steel without the TENG is about –0.73 V (*vs.* SCE) and shifts sharply to –0.99 V (*vs.* SCE) after being connected to the cathodic protection system powered by the TENG. Therefore, the OCP shifts negatively by about 260 mV, indicating that the carbon steel is under effective cathodic protection. It is noteworthy that when the TENG is removed, the OCP shifts positively and recovers to the position near to the original value. The periodic change of the OCP reflects the high repeatability of the PPy NW-based TENG for powering the cathodic protection system.

In order to directly observe the cathodic protection effect powered by the PPy-based TENG, polished carbon steels connected with and without the PPy-based TENG have been immersed into 3.5 wt% NaCl solution for 2 h, 4 h, and 6 h, respectively. Their microscopy images are shown in Fig. 6c. It is obvious that the surface of the carbon steel is covered completely by red rust after immersion for 2 h and becomes increasingly worse with prolonging of immersion time. However, when the PPy-based TENG is connected to the protected carbon steel, there is no apparent corrosion occurring after immersion for 2 h and only a few corrosion pits are found on the surface with the immersion time extended to 6 h. As a result, the carbon steel connected with the PPy-based TENG is protected effectively from corrosion. This is because the electrons generated during the operation of the TENG would flow through the connecting wire and finally be injected into the surface of the carbon steel to form cathodic protection. Therefore, the cathodic protection system powered by the TENG is an effective electrochemical means of corrosion control in which the oxidation reaction is mainly taken at the anode and corrosion of the cathode, *i.e.* the protected carbon steel, is suppressed at the same time.

**Fig. 7:**
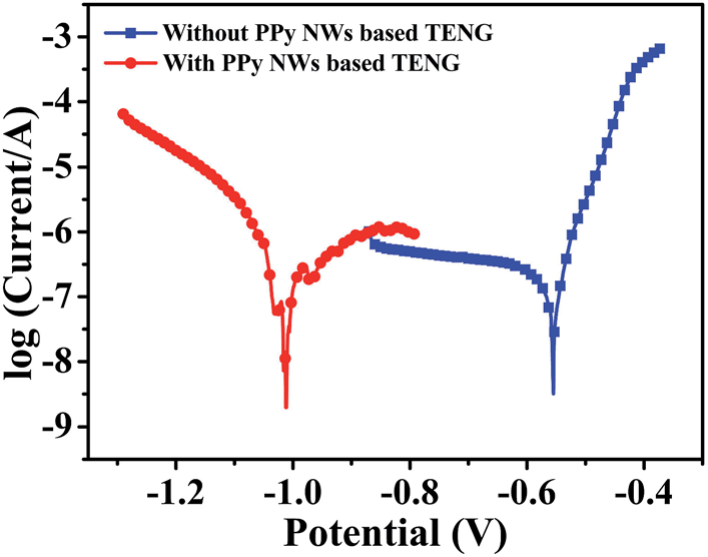
Polarization curves of Q235 carbon steel connected with and without the PPy NW-based TENG.

Furthermore, electrochemical polarization curves, as one of the common means to evaluate corrosion behaviour, have been measured. Corrosion potential (*E*_corr_) and corrosion current (*I*_corr_) can be estimated by extrapolating the linear Tafel regions on the cathodic and anodic branches. In the case of the cathodic protection system, a negative shift in *E*_corr_ shows a better cathodic protection effect on corrosion resistance.^44^ Here, the polarization curves of the carbon steel connected with and without the PPy-based TENG are shown in Fig. 7. The corresponding values of the electrochemical parameters, including *E*_corr_, *I*_corr_, anodic Tafel slope (*β*_a_) and cathodic Tafel slope (*β*_c_), are analysed from the polarization curves in Table 1. When connected with the cathodic protection system powered by the TENG, the *E*_corr_ of the carbon steel shows a negative shift, which agrees well with the OCP drop in Fig. 6b. Meanwhile, *I*_corr_ shows a slight increase because the injection of electrons produced by the TENG enhances the electrochemical reaction at the surface of the carbon steel.^53^ Therefore, these results of the polarization curves indicate that the TENG as the power supplier of the cathodic protection system can protect the carbon steel from corrosion by providing sufficient electrons.

**Table 1:** Electrochemical parameters obtained from polarization curves of Q235 carbon steel connected with and without the PPy NW-based TENG

	*E*_corr_ (V (*vs.* SCE))	*I*_corr_ (μA)	–*β*_c_ (mV dec^–1^)	*β*_a_ (mV dec^–1^)
Without TENG	–0.544	25.91	874	38.8
With TENG	–0.973	70.79	165	525

The cathodic protection measurement was also driven by the PPy NW-based W-TENG, with its structure shown in Fig. 5a, under a wind speed of 7 m s^–1^. The device structure of the cathodic protection system powered by the PPy NW-based W-TENG is shown in Fig. S1.† Relevant data including microscopy images, OCP variation and Tafel curves of the Q235 carbon steel are given in the ESI.† Fig. S2† shows the microscopy images of the Q235 carbon steel powered by PPy NW-based W-TENG for different times. It can be seen that nearly no obvious corrosion is observed with the immersion time extended to 6 h. The negative shift of the OCP (Fig. S3†) and *E*_corr_ (Fig. S4†) also prove that the Q235 carbon steel can be effectively protected from corrosion by the cathodic protection system powered by the PPy NW-based W-TENG at a low wind speed.

## Conclusions

In summary, a novel PPy NW-based TENG is successfully constructed with PPy NWs prepared by an electrochemical polymerization method with AAO as the template. It turns out that the PPy NW-based TENG shows good durability and high output performance with a short circuit current density of 23.4 mA m^–2^ and output voltage of 351 V. Moreover, instead of the external power source in the traditional impressed cathodic protection system, the PPy NW-based TENG, driven by a linear mechanical motor to simulate mechanical energy, can serve as a power supplier and provide sufficient electrons to the surface of protected Q235 carbon steel. The electrochemical tests of OCP and polarization curve measurements showed that the carbon steel could be protected from corrosion when connected with the cathodic protection system powered by the TENG. In addition, another PPy NW-based W-TENG designed for harvesting wind energy has also exhibited good output performance at a low wind speed and could provide sufficient charges to the cathodic protection system. The PPy NW-based TENG has great potential for application in the cathodic protection system by converting different kinds of energies into electricity, due to its advantages of low cost, simple preparation, portability, and so on.

## Experimental

### Materials

Pyrrole (Aladdin) was purified by distillation prior to use and stored at low temperature under a nitrogen atmosphere. The commercial AAO templates with an average nanopore diameter of around 100 nm and thickness of 100 μm were purchased from Puyuan Nano Technology Co. Ltd. (Hefei, China). Lithium perchlorate trihydrate (LiClO_4_) (Aladdin), PVDF (Aldrich) and other solvents were used without further treatment.

### Preparation of PPy NWs

PPy NWs were prepared on Ti substrate by an electrochemical polymerization method with AAO as the template in a two-electrode cell with 0.1 M LiClO_4_ and 0.2 M pyrrole acetonitrile solution as the electrolyte. Before electropolymerization, the AAO template was fixed on the Ti substrate using epoxy resin with an exposed electrode area of 12 cm^2^, and then immersed into the electrolyte and vacuumed to remove the residual air bubbles in the AAO nanopores. Ti foil fixed with AAO template was used as the anode and Pt foil as the cathode. The electropolymerization was performed at a constant potential of 3 V for 1 min. After that, the as-prepared sample was rinsed carefully with distilled water. Finally, the AAO template was dissolved in 2 M NaOH at room temperature for 12 h and PPy NWs grown on Ti substrate were obtained.

### Fabrication of PPy NW-based TENG

To fabricate the PPy NW-based TENG as shown in Fig. 1b, PPy NWs grown on Ti substrate acted as one of the triboelectrodes and the other electrode was made by spin-coating PVDF on a Kapton film (0.05 mm thickness, 12 cm^2^). Briefly, 3.75 g PVDF powder was dissolved in a mixed solution of 8.5 g *N*,*N*-dimethylacetamide (DMAC) and 12.75 g acetone by stirring at 60 °C. Then, the PVDF solution was spin-coated on a Kapton film at 3000 rpm for 30 s. After that, a piece of Cu tape was pasted on the back of the Kapton film, with a Cu wire connected to the surface of the Cu tape by conductive silver epoxy. The TENG based on PPy NW and PVDF triboelectrodes was driven by a commercial linear mechanical motor under the contact-separation mode with a total contact area of 12 cm^2^.

### Fabrication of PPy NW-based W-TENG

The as-prepared PPy NWs on Ti substrate were used as one of the triboelectrodes. A highly flexible PE thin-film with a thickness of 10 μm was selected as the substrate of the other triboelectrode. One side of the PE film was sputtered with Ag by magnetron sputtering, and the other side of the PE film was deposited with PVDF nanofibers by the electrospinning method. Then, the PVDF-based triboelectrode was fixed on the four edges of the PPy NW-based triboelectrode with acrylic sheet as the spacer (thickness of 1 mm). Wind was simulated by a commercial air gun, and wind speeds were measured by a digital anemometer.

### Cathodic protection system powered by TENG and W-TENG

Q235 carbon steel electrodes were mechanically ground with SiC papers successively down to 1500 grit to remove the oxide layer, and rinsed with acetone, ethanol and distilled water, respectively. In the cathodic protection system, the protected metal was connected to the negative electrode of the rectified TENG while Pt foil as the counter electrode was connected to the positive electrode. The simulated corrosion tests with and without TENG or W-TENG were performed in 3.5 wt% NaCl solution for comparison and the carbon steel electrodes were taken out and tested for the degree of corrosion after being immersed for 2 h, 4 h and 6 h, respectively.

### Characterization

The morphology of the PPy NWs was observed by field emission scanning electron microscopy (FESEM, JSM-6701F, JEOL Inc., Japan). The performance of the PPy NW-based TENG, including *I*_sc_ and *V*_o_, was measured *via* a SR570 low noise current preamplifier (Stanford Research Systems) and NI-PCI6259 (National Instruments). The *V*_o_ was measured with a load resistor of 100 MΩ. The degree of corrosion of the carbon steel was observed using an optical microscope (Olympus BX51, Japan). The OCP variation of the carbon steel coupled with and without the TENG or W-TENG was tested with an electrochemical workstation (CHI 660E, Shanghai Chenhua Instrument Co., Ltd, China) in a three-electrode system with SCE as the reference electrode and Pt foil as the counter electrode.
