# Supplementary material for: Conducting polymer PPy nanowire-based triboelectric nanogenerator and its application for self-powered electrochemical cathodic protection
Source: Chem Sci. 2016 Jun 27;7(10):6477–83. doi: 10.1039/c6sc02562e (PMC5356033; doi:10.1039/c6sc02562e)
Supplement: Supplementary file 1 [file SC-007-C6SC02562E-s001.pdf]

Electronic Supplementary Material (ESI) for Chemical Science.
This journal is © The Royal Society of Chemistry 2016

Supporting Information:

Conducting polymer PPy nanowires based triboelectric nanogenerator and its application for self-powered electrochemical cathodic protection

Siwen Cui[a,b], *Youbin Zheng*[a], *Jun Liang*[a], *Daoai Wang*[a,]*

[a]State Key Laboratory of Solid Lubrication, Lanzhou Institute of Chemical Physics, Chinese Academy of Sciences, Lanzhou 730000, China;

[b]University of Chinese Academy of Sciences, Beijing, 100049, China

*Corresponding Author, E-mail: wangda@licp.cas.cn

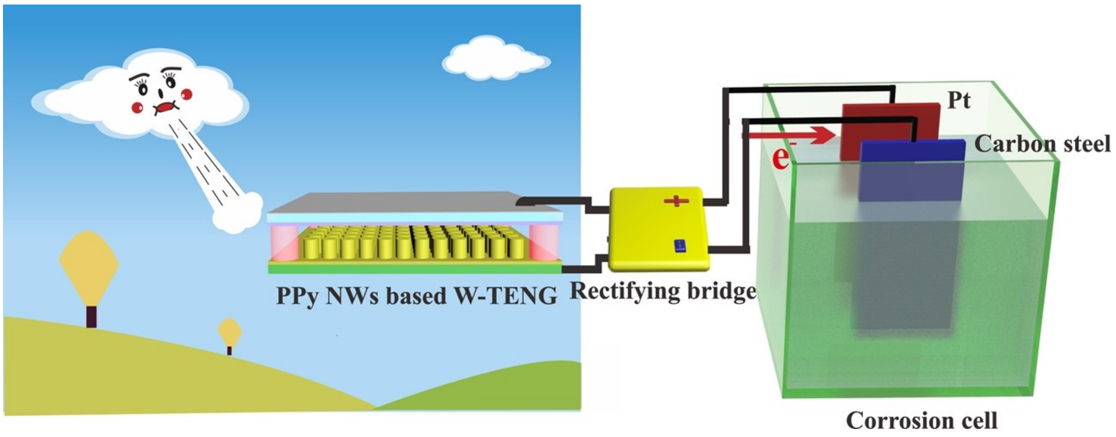

Figure S1. The device structure of the cathodic protection system powered by PPy NWs based W-TENG.

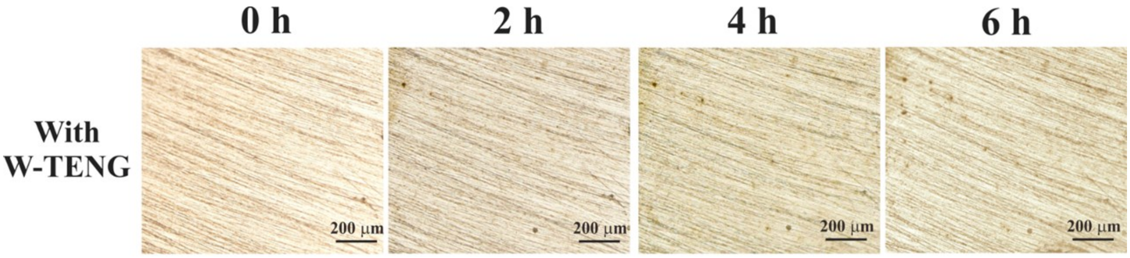

Figure S2. Microscope images of the Q235 carbon steel immersed in 3.5 wt % NaCl solution for 2 h, 4 h and 6 h, separately, which connected with PPy NWs based W-TENG.

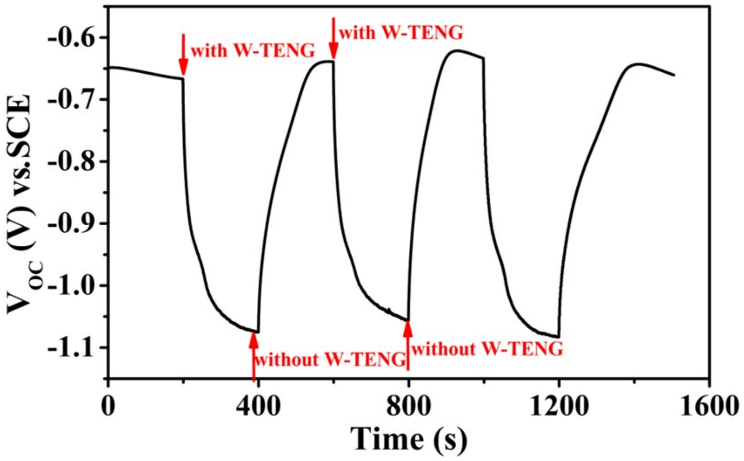

Figure S3. OCP changes of Q235 carbon steel coupled with and without the PPy NWs based W-TENG.

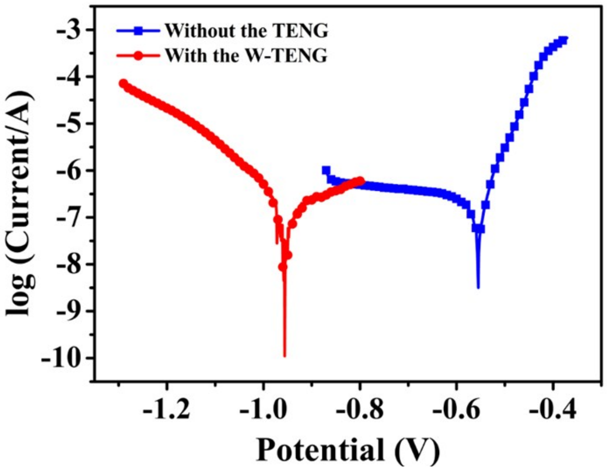

Figure S4 Polarization curves of Q235 carbon steel connected with and without the PPy NWs based W-TENG.
